# A structure determination protocol based on combined analysis of 3D-ED data, powder XRD data, solid-state NMR data and DFT-D calculations reveals the structure of a new polymorph of l-tyrosine†

**DOI:** 10.1039/d1sc06467c

**Published:** 2022-03-30

**Authors:** Christopher J. H. Smalley, Harriet E. Hoskyns, Colan E. Hughes, Duncan N. Johnstone, Tom Willhammar, Mark T. Young, Christopher J. Pickard, Andrew J. Logsdail, Paul A. Midgley, Kenneth D. M. Harris

**Affiliations:** School of Chemistry, Cardiff University Park Place Cardiff CF10 3AT Wales UK HarrisKDM@cardiff.ac.uk; Department of Materials Science, University of Cambridge 27 Charles Babbage Road Cambridge CB3 0FS England UK; Department of Materials and Environmental Chemistry, Stockholm University Svante Arrhenius väg 16C 106 91 Stockholm Sweden; School of Biosciences, Cardiff University Cardiff CF10 3AX Wales UK; Advanced Institute for Materials Research, Tohoku University 2-1-1 Katahira Aoba Sendai 980-8577 Japan; Cardiff Catalysis Institute, School of Chemistry, Cardiff University Park Place Cardiff CF10 3AT Wales UK

## Abstract

We report the crystal structure of a new polymorph of l-tyrosine (denoted the β polymorph), prepared by crystallization from the gas phase following vacuum sublimation. Structure determination was carried out by combined analysis of three-dimensional electron diffraction (3D-ED) data and powder X-ray diffraction (XRD) data. Specifically, 3D-ED data were required for reliable unit cell determination and space group assignment, with structure solution carried out independently from both 3D-ED data and powder XRD data, using the direct-space strategy for structure solution implemented using a genetic algorithm. Structure refinement was carried out both from powder XRD data, using the Rietveld profile refinement technique, and from 3D-ED data. The final refined structure was validated both by periodic DFT-D calculations, which confirm that the structure corresponds to an energy minimum on the energy landscape, and by the fact that the values of isotropic ^13^C NMR chemical shifts calculated for the crystal structure using DFT-D methodology are in good agreement with the experimental high-resolution solid-state ^13^C NMR spectrum. Based on DFT-D calculations using the PBE0-MBD method, the β polymorph is meta-stable with respect to the previously reported crystal structure of l-tyrosine (now denoted the α polymorph). Crystal structure prediction calculations using the AIRSS approach suggest that there are three other plausible crystalline polymorphs of l-tyrosine, with higher energy than the α and β polymorphs.

## Abstract 2

A new polymorph of l-tyrosine is reported, with the crystal structure determined by combined analysis of 3D-ED data and powder XRD data, augmented by information from periodic DFT-D calculations and solid-state ^13^C NMR data.

## Graphical abstract

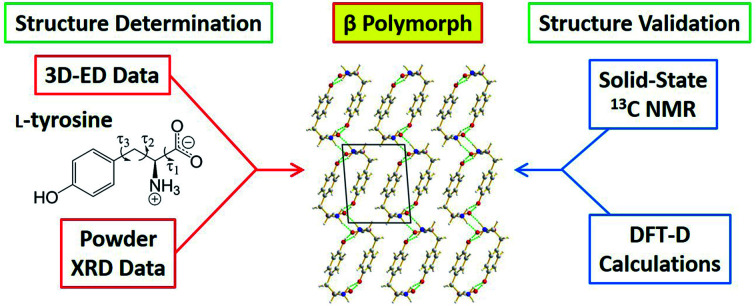

## Introduction

For many materials, it is difficult to prepare single-crystal specimens of suitable size to allow the measurement of single-crystal X-ray diffraction (XRD) data of adequate quality for crystal structure determination. Under such circumstances, crystal structure determination must rely on the use of other experimental approaches that allow diffraction data to be recorded on microcrystalline samples. One approach is to carry out structure determination directly from powder XRD data.^1–9^ However, in spite of continuing advances in data measurement techniques and data analysis strategies, successful structure determination from powder XRD data may be rendered difficult or impossible due to certain intrinsic challenges that originate from the nature of the powder sample itself, for example: (i) if the powder sample contains two or more different crystalline phases (*e.g.*, containing an unknown impurity phase together with the main phase of interest), the process of unit cell determination may be very difficult or impossible, and (ii) if the powder sample exhibits a non-random distribution of crystallite orientations (so-called “preferred orientation”), the relative intensities of peaks in the experimental powder XRD data may differ significantly from the intrinsic relative diffraction intensities characteristic of the crystal structure – as a consequence, achieving successful structure solution from the powder XRD data may face insurmountable challenges.

An alternative approach that circumvents both of these problems is to record three-dimensional electron diffraction (3D-ED) data on individual micro-crystals within the powder sample, effectively yielding single-crystal diffraction data for the selected micro-crystals. Recent progress in the development of instrumentation and data collection protocols has facilitated the measurement of 3D-ED data suitable for use in crystal structure determination calculations.^10–20^ Nevertheless, the 3D-ED approach also suffers from certain intrinsic challenges, including the susceptibility of materials to undergo beam damage upon electron beam irradiation. This may result in data of low resolution and/or low completeness, which can hamper structure solution and refinement. Furthermore, intensities obtained using electron diffraction will be affected by dynamical scattering. Although structure solution and refinement based on the kinematical scattering approximation are able to deliver a structural model that is a good representation of the crystal structure,^21^ obtaining a more accurate description of the crystal structure requires more rigorous consideration of the effects of dynamical scattering.^15,16,19^

Based on consideration of the various challenges discussed above, an attractive strategy for crystal structure determination of micro-crystalline materials is to record *both* 3D-ED data *and* powder XRD data, and then (i) to tackle unit cell determination using the 3D-ED data, (ii) to carry out structure solution using the 3D-ED data or the powder XRD data (or, ideally, using both datasets independently), and (iii) to achieve high-quality structure refinement using the powder XRD data (ensuring, *inter alia*, that all XRD-detectable phases in the micro-crystalline powder sample are properly taken into account). In the present paper, we demonstrate the successful application of this strategy to determine the structure of a new polymorph of l-tyrosine. Two other components of the strategy used in this work are: (i) to apply periodic DFT-D calculations to improve the quality of the structural model at various stages during the structure determination process, including to provide a rigorous validation^22–26^ of the final structure obtained in Rietveld refinement from powder XRD data, and (ii) to assess the agreement between calculated solid-state NMR data, computed by DFT-D methodology for the final structure obtained in Rietveld refinement from the powder XRD data, and the corresponding experimental solid-state NMR data, again as part of the structure validation process.^26–34^

Among the 20 directly-encoded proteinogenic amino acids in their natural (enantiomerically pure) form, each member of this set has now had a crystal structure reported, following recent structure determinations of l-arginine,^35^l-tryptophan^36,37^ and l-lysine^38^ [we note that one member (glycine) of this set is non-chiral, while the other 19 amino acids are chiral and exist in nature only as the l-enantiomer]. Polymorphism is also known for some of these biologically important materials, with structurally characterized polymorphs reported for l-cysteine, l-glutamic acid, glycine, l-histidine, l-isoleucine, l-leucine, l-phenylalanine, l-proline, l-serine and l-tryptophan.

**Fig. 1:**
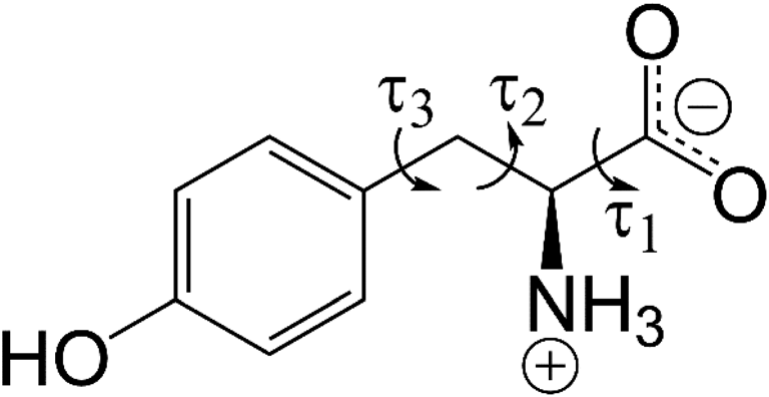
Molecular structure of l-tyrosine in the zwitterionic form. The variable torsion angles (*τ*_1_, *τ*_2_ and *τ*_3_) in the direct-space GA structure solution calculations are indicated.

In the present paper, we report the preparation and structural characterization of a new polymorph of l-tyrosine (Fig. 1) obtained by crystallization from the gas phase, with structure determination carried out using the protocol outlined above, involving combined analysis of 3D-ED data and powder XRD data in conjunction with periodic DFT-D calculations, and with final structure validation also based on consideration of the agreement between calculated and experimental solid-state NMR data. Furthermore, the polymorphic landscape of l-tyrosine has been explored through crystal structure prediction calculations,^39–44^ specifically using the AIRSS method.^45,46^ The crystal structure of l-tyrosine determined previously (from single-crystal XRD data^47^ and neutron diffraction data^48^) is now denoted the α polymorph, and the new polymorph reported here is denoted the β polymorph.

## Results and discussion

### Structure determination of the β polymorph of l-tyrosine

The new polymorph of l-tyrosine was prepared by crystallization from the gas phase following sublimation of a sample of the α polymorph (see *Methods*), and was clearly identified as a new solid form based on the fact that the powder XRD pattern is significantly different from that characteristic of the α polymorph. Evidence has been reported previously^49^ for a new solid form of l-tyrosine prepared by this approach, but the crystal structure was not determined in the previous study (after completing the work in the present paper, it was apparent from the powder XRD data reported previously that the sample prepared by crystallization from the gas phase in the previous work^49^ was a mixture of the α and β polymorphs).

Our initial attempts to progress with structure determination of the new polymorph from powder XRD data were unsuccessful as indexing (unit cell determination) using algorithms in the CRYSFIRE suite^50^ could not account for all peaks present in the powder XRD pattern. However, 3D-ED data recorded for the same sample were indexed successfully using REDp^51^ and subsequently XDS^52^ to give the following unit cell with monoclinic metric symmetry: *a* = 7.92 Å, *b* = 6.13 Å, *c* = 9.90 Å, *β* = 94.82°, *V* = 478.9 Å^3^. The space group was assigned from the 3D-ED data as *P*2_1_, based on systematic extinctions. Given the volume of this unit cell, density considerations suggest that there are two molecules in the unit cell and therefore one molecule in the asymmetric unit for space group *P*2_1_.

The unit cell determined from the 3D-ED data shed light on the failure to index the powder XRD data. Most peaks in the powder XRD data were consistent with the unit cell determined from the 3D-ED data, but the powder XRD data also contained a few additional peaks due to a second crystalline phase (identified as the α polymorph of l-tyrosine; Fig. S2†). Clearly, the biphasic nature of the powder sample was the reason for the failure to index the powder XRD data in this case (however, we note that the presence of a minor phase in a biphasic powder sample does not always prevent successful unit cell determination of the major phase in attempting to index the powder XRD data; in favourable cases, it can be possible to index the peaks due to the major phase in a biphasic powder sample, as demonstrated in previous studies^37^).

Structure solution of the new polymorph of l-tyrosine was carried out using the 3D-ED data, as described below. Furthermore, although the powder XRD data contained additional peaks due to a second phase, the number and intensities of these peaks were relatively low, such that it was considered viable also to attempt structure solution from the powder XRD data (encouraged by previous cases in which structure solution from powder XRD data containing a small number of peaks due to an impurity phase were successful^37^). Thus, structure solution was also attempted from the powder XRD data.

Structure solution was carried out independently from the 3D-ED data and powder XRD data using the direct-space strategy implemented using a genetic algorithm (GA) in the program EAGER.^53–56^ While EAGER was originally developed and applied for structure solution from powder XRD data,^57–63^ it has been extended recently to allow structure solution from 3D-ED data within the kinematical scattering approximation.^64^ In the direct-space GA structure solution calculations, the contents of the asymmetric unit comprised one molecule of l-tyrosine in the zwitterionic form, constructed using standard bond lengths and bond angles (based on geometric information from MOGUL^65^ and, for bonds involving hydrogen, from Allen *et al.*^66^). Trial crystal structures were defined by a total of eight structural variables (two positional, three orientational and three torsional variables; the torsion-angle variables are defined in Fig. 1). We note that, for space group *P*2_1_, the position of the molecule along the *b*-axis can be fixed; thus, only two positional variables are required in the direct-space search.

**Fig. 2:**
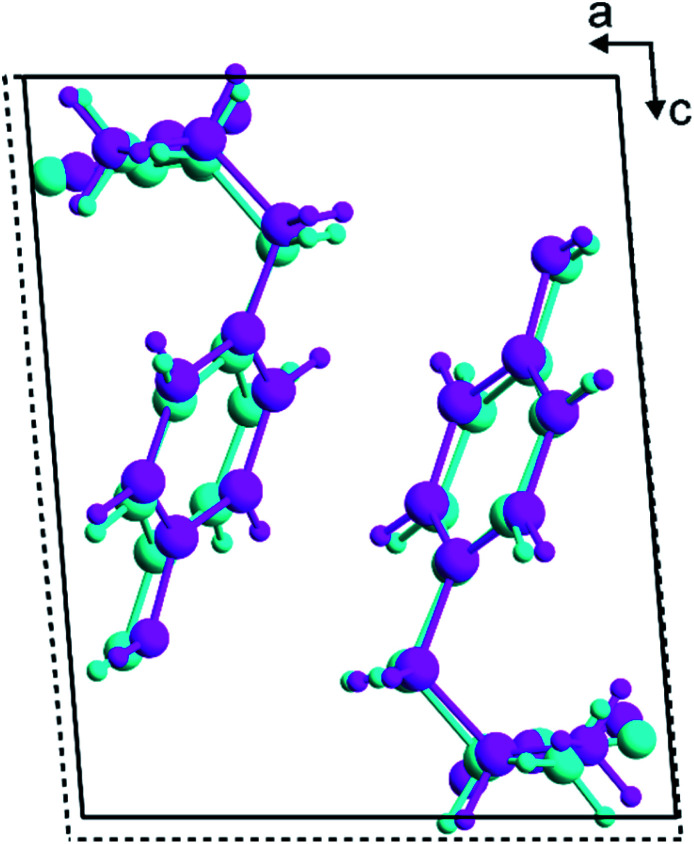
Overlay of the best structure solutions (with lowest *R*-factor) viewed along the *b*-axis for the β polymorph of l-tyrosine obtained from the direct-space GA structure solution calculations using 3D-ED data (cyan; unit cell shown by the dashed black lines) and powder XRD data (magenta; unit cell shown by the solid black lines).

In the structure solution calculations using the 3D-ED data and the powder XRD data (for the original biphasic sample), a total of 40 independent GA structure solution calculations were carried out in each case from different random initial populations. Each GA calculation involved the evolution of a population of 100 trial structures for 100 generations, with 10 mating operations and 50 mutation operations carried out per generation. The *R*-factors used to assess the quality of trial structures (based on the level of agreement between calculated and experimental diffraction data) in the structure solution calculations from the 3D-ED data (*R*_F_) and powder XRD data (*R*_wp_) are defined in Section S4,† and the evolution of lowest *R*-factor in the population as a function of generation number in the 40 independent GA calculations using the 3D-ED data and the powder XRD data are shown in Fig. S4.† Using the 3D-ED data, 7 of the 40 independent GA calculations generated essentially the same structure solution with lowest *R*_F_, whereas using the powder XRD data, 38 of the 40 independent GA calculations generated essentially the same structure solution with lowest *R*_wp_ (Fig. S4†). The best structure solutions (with lowest *R*-factor) from the 3D-ED data and the powder XRD data represent essentially the same structure (Fig. 2), with root-mean-squared difference (RMSD) of only 0.33 Å between the positions of the non-hydrogen atoms in these structures. The best structure solution from the powder XRD data was used as the starting structural model for Rietveld refinement,^67^ which was carried out using TOPAS.^68^

**Fig. 3:**
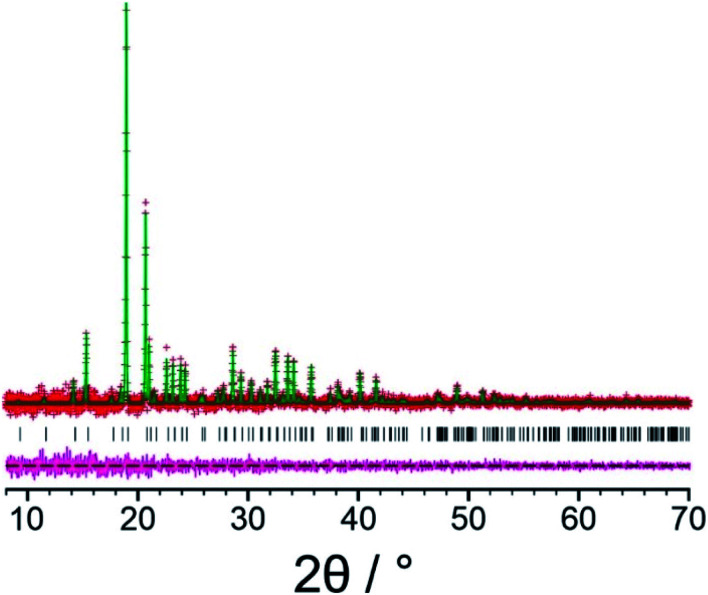
Profile fitting (using the Pawley method) of the powder XRD data (background subtracted) for the β polymorph of l-tyrosine, showing the experimental powder XRD data (red “+” marks), the calculated powder XRD data (green line), the predicted peak positions (black tick marks) and the difference between experimental and calculated powder XRD data (magenta line).

**Fig. 4:**
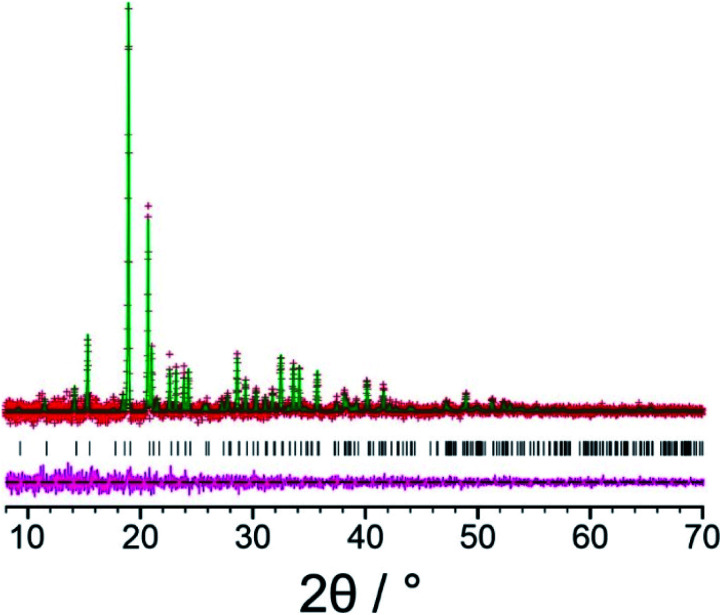
Final Rietveld refinement of the powder XRD data (background subtracted) for the β polymorph of l-tyrosine, showing the experimental powder XRD data (red “+” marks), the calculated powder XRD data (green line), the predicted peak positions (black tick marks) and the difference between experimental and calculated powder XRD data (magenta line).

Our initial Rietveld refinement used the powder XRD dataset for the original biphasic sample that was used in the structure solution calculations described above. However, at this stage, further attempts to prepare the new polymorph of l-tyrosine by crystallization from the gas phase under different experimental conditions (see *Methods*) were successful in producing a monophasic sample, confirmed by powder XRD. A high-quality powder XRD dataset for this monophasic sample was used for the final Rietveld refinement. Initially, profile fitting using the Pawley method^69^ gave a good fit to the experimental powder XRD data (Fig. 3; *R*_wp_ = 0.29%, *R*_p_ = 0.22%). The structure solution from the EAGER calculations on the powder XRD data discussed above (Fig. 2) was used as the initial structural model for the Rietveld refinement, with standard restraints applied to bond lengths and bond angles, and planar restraints applied to the phenyl ring and carboxylate group. A common isotropic displacement parameter was refined for all non-hydrogen atoms, and the isotropic displacement parameter for hydrogen atoms was set at 1.2 times this value. As powder XRD data recorded for the same powder sample using a two-dimensional detector (Fig. S3†) clearly showed non-uniform intensity of the Debye–Scherrer rings, it was concluded that the powder sample exhibits preferred orientation; thus, a preferred orientation correction was applied in the Rietveld refinement using the March–Dollase method,^70,71^ with a refined March parameter of 0.67 for the (0 1 0) plane. After completing the Rietveld refinement, the refined structure was subjected to DFT-D geometry optimization with fixed unit cell, which led to only minor structural changes (we emphasize that, in utilizing DFT-D geometry optimization in this way to facilitate improvements in the structural model in tandem with Rietveld refinement, it is crucial that the unit cell is fixed in the DFT-D geometry optimization calculations in order that the unit cell in the geometry-optimized structure matches the unit cell corresponding to the experimental powder XRD data). The geometry optimized structure was then used as the starting model for the final Rietveld refinement, which included additional restraints on the geometries of hydrogen bonds based on the structure obtained in the DFT-D geometry optimization. The final Rietveld refinement gave a good-quality fit to the powder XRD data (Fig. 4; *R*_wp_ = 0.30%, *R*_p_ = 0.23%), comparable to the quality of fit obtained in Pawley fitting (Fig. 3), with the following final refined parameters: *a* = 7.6764(6) Å, *b* = 5.8870(3) Å, *c* = 9.6143(6) Å, *β* = 94.575(4)°, *V* = 433.10(5) Å^3^ (2*θ* range, 8°–70°; 3858 profile points; 93 refined variables; 81 restraints). The crystal structure of the β polymorph of l-tyrosine obtained in the final Rietveld refinement has been deposited in the CSD (deposition number: 2114085).

Least-squares refinement was also carried out against the 3D-ED data using the kinematical treatment. The starting structural model for the refinement was the structure solution obtained from the 3D-ED data using EAGER (see Fig. 2), with unit cell parameters taken from the profile-fitting (in this case Pawley fitting) of the powder XRD data and with hydrogen atoms included based on geometric restraints. An initial refinement using isotropic atomic displacement parameters converged with *R*1 = 25.1%. A final refinement using additional restraints on the C–C, C–O and C–N distances in order to improve the bonding geometries converged with *R*1 = 25.1%. The final refined structures from the powder XRD data and 3D-ED data are in good agreement (Fig. S5;† RMSD for non-hydrogen atoms, 0.135 Å).

**Fig. 5:**
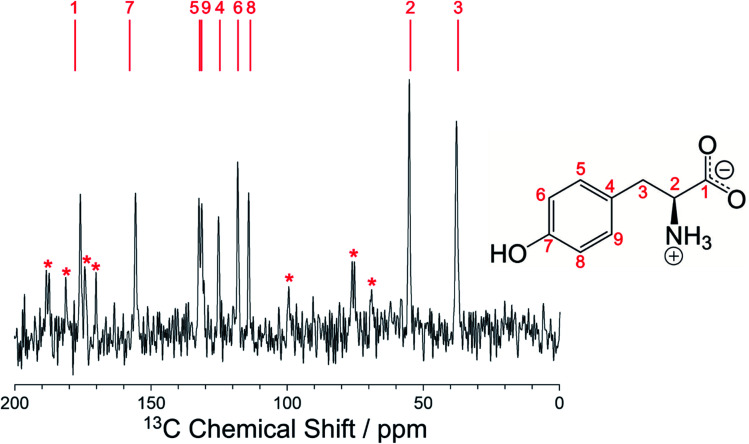
Experimental high-resolution solid-state ^13^C NMR spectrum recorded for the β polymorph of l-tyrosine together with the values of isotropic ^13^C NMR chemical shifts calculated for the crystal structure of the β polymorph (indicated by the red lines above the spectrum). The specific ^13^C site corresponding to each calculated value is indicated. Spinning sidebands in the experimental spectrum are marked by red asterisks.

As further validation of the crystal structure of the β polymorph of l-tyrosine, DFT-D geometry optimization (using PBE-TS and with fixed unit cell) on the final refined structure from the Rietveld refinement led to only minor atomic displacements (Fig. S6;† RMSD for non-hydrogen atoms, 0.025 Å), confirming that the final refined crystal structure is very close to a minimum on the energy landscape. Furthermore, the isotropic solid-state ^13^C NMR chemical shifts calculated using DFT-D/GIPAW methodology (see *Methods*) for the crystal structure of the β polymorph (Table S5†) are in close agreement with the isotropic chemical shifts in the experimental high-resolution solid-state ^13^C NMR spectrum of this material, as shown in Fig. 5. Thus, in addition to being in excellent agreement with the experimental powder XRD data, as presented above (Fig. 4), our reported crystal structure of the β polymorph of l-tyrosine is also confirmed to be in excellent agreement with the experimental solid-state ^13^C NMR data.

We note that the high-resolution solid-state ^13^C NMR spectrum of the β polymorph (Fig. 5) is clearly distinct from that of the α polymorph (Fig. S10†), and in each case there is good agreement between the experimental solid-state ^13^C NMR spectrum and the isotropic ^13^C NMR chemical shifts calculated from the crystal structure using DFT-D/GIPAW methodology (Table S5†). These observations highlight the utility of DFT-D/GIPAW calculations to compute reliable solid-state NMR data for known crystal structures, both in the context of polymorph characterization and as a powerful strategy in conjunction with structure determination from powder XRD data.

### Relative energies of the α and β polymorphs of l-tyrosine

To assess the relative energetic properties of the crystal structure of the β polymorph of l-tyrosine reported here and the crystal structure of the α polymorph reported previously^47^ (from single-crystal XRD), periodic DFT-D calculations (see *Methods*) were carried out using different exchange-correlation functionals (PBE and PBE0) combined with different methods for dispersion correction (TS and MBD). For each polymorph, the experimentally determined crystal structure at ambient temperature was taken as the initial structural model for DFT-D geometry optimization (with fixed unit cell) using PBE-TS. For the resulting geometry optimized structures, single-point energy calculations were carried out using each of the four combinations of functional and dispersion correction method discussed above. For each calculation method, the energy is higher for the β polymorph than the α polymorph, with the following energy differences (expressed per mole of l-tyrosine molecules): 4.74 kJ mol^−1^ (PBE-TS), 3.96 kJ mol^−1^ (PBE-MBD), 3.66 kJ mol^−1^ (PBE0-TS), 3.64 kJ mol^−1^ (PBE0-MBD). Among these methods, PBE0-MBD is considered^72,73^ to give the most reliable assessment of the relative energies of polymorphs of organic materials. We note that the calculated energy difference using PBE0-MBD of 3.64 kJ mol^−1^ between the α and β polymorphs is fully consistent^74^ with the β polymorph being an experimentally observable meta-stable polymorph of l-tyrosine. From the crystal structures determined at ambient temperature, the densities of the α and β polymorphs are 1.414 g cm^−3^ and 1.389 g cm^−3^, respectively. Clearly, the higher density of the α polymorph may be a contributing factor to the lower energy of this polymorph established from the DFT-D calculations.

### Structural properties of the β polymorph of l-tyrosine

**Fig. 6:**
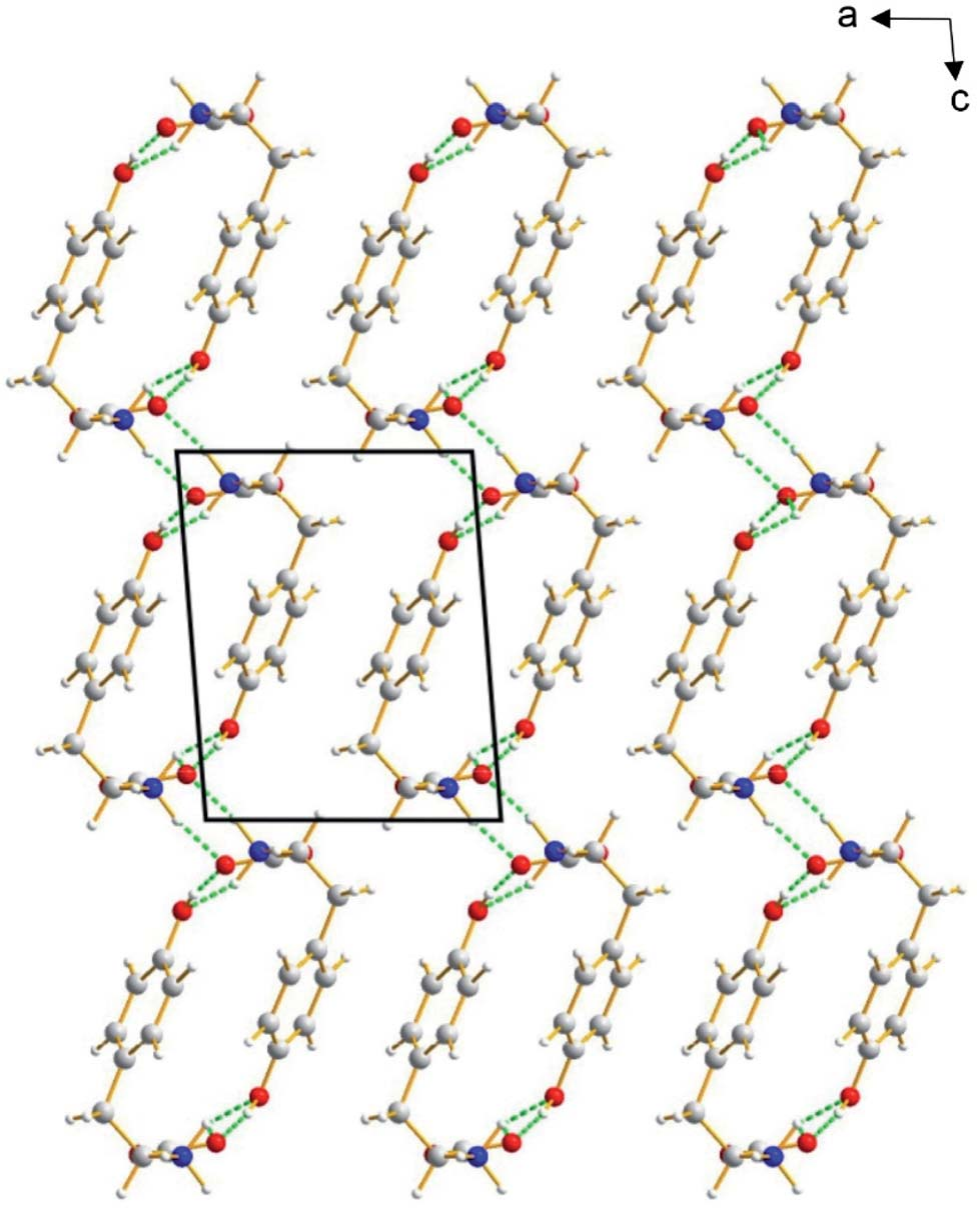
Crystal structure of the β polymorph of l-tyrosine viewed along the *b*-axis, showing the alternating hydrophilic and hydrophobic layers parallel to the *ab*-plane (horizontal). The hydrogen-bonded ribbons involving the amino-acid head-groups propagate along the direction of view. Hydrogen bonds are indicated by green dashed lines.

**Fig. 7:**
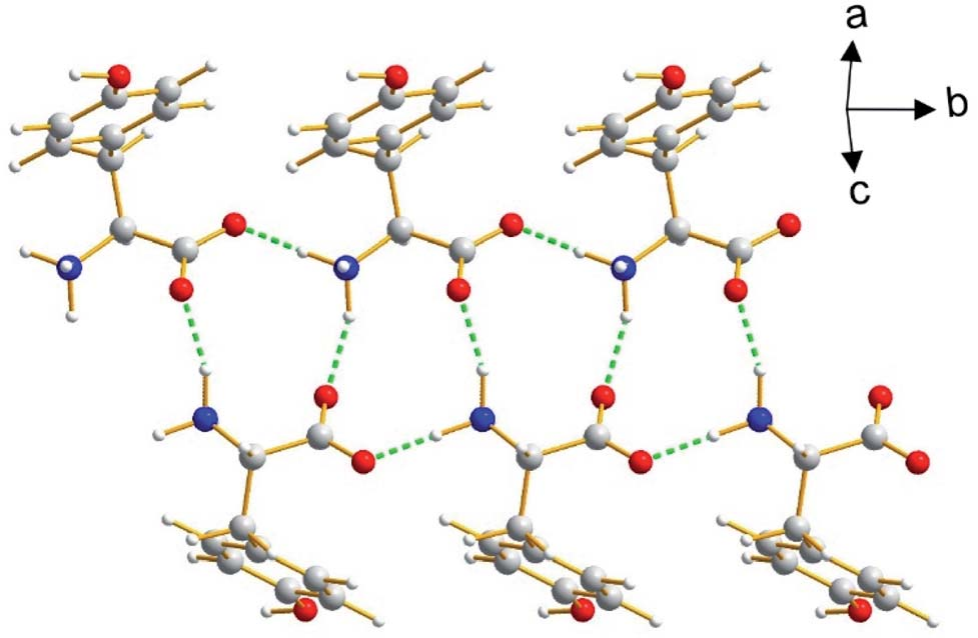
The hydrogen-bonded ribbon involving the amino acid head-groups in the β polymorph of l-tyrosine. Hydrogen bonds are indicated by green dashed lines.

**Fig. 8:**
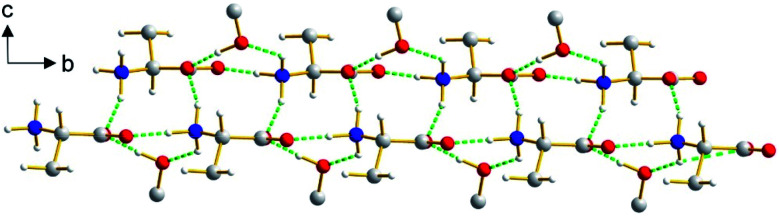
Hydrogen bonding between the ribbon containing the amino acid head-groups and the OH groups of the side-chains (from molecules above and below the ribbon shown) in the β polymorph of l-tyrosine. For clarity, only the CH_2_CH(NH_3_^+^)CO_2_^−^ unit of each head-group and only the COH unit of each side-chain are shown. The two strands of the ribbon propagate along the *b*-axis (horizontal) and are offset slightly from each other along the *c*-axis (vertical). Hydrogen bonds are indicated by green dashed lines.

The β polymorph of l-tyrosine (Fig. 6) comprises alternate hydrophobic and hydrophilic layers parallel to the *ab*-plane. The hydrophilic region contains the amino acid head-groups and the OH groups of the side-chains, while the hydrophobic region contains the phenyl rings of the side-chains. The hydrogen-bonding involving the amino acid head-groups gives rise to a ribbon motif (Fig. 7) propagating along the *b*-axis. The ribbon is constructed from two strands of l-tyrosine molecules, with N–H⋯O hydrogen bonding between adjacent molecules in a given strand and between molecules in the two strands. The ribbon is also engaged in hydrogen bonding with the OH groups of the side-chains of the molecules that form the hydrogen-bonded ribbons in the layers “above” and “below” along the *c*-axis (Fig. 8). The contiguous hydrogen-bonded network comprises corrugated slabs (with a mean plane parallel to the *bc*-plane; Fig. 6) constructed from the hydrogen-bonded ribbons parallel to the *b*-axis and hydrogen-bonding (involving the OH groups) to adjacent ribbons along the *c*-axis. Adjacent corrugated slabs are related by translation along the *a*-axis and interact through van der Waals interactions.

We now compare the structural properties of the α and β polymorphs, firstly noting that the molecular conformation is similar in each polymorph. However, while the β polymorph contains one-dimensional hydrogen-bonded ribbons, the hydrophilic region of the α polymorph comprises a two-dimensional hydrogen-bonded array (Fig. S8 and S9†) involving the amino acid head-groups and the OH groups of molecules in the layers above and below. Consequently, the α polymorph is a three-dimensionally connected hydrogen-bonded structure, in contrast to the two-dimensionally connected hydrogen-bonding arrangement (corrugated slabs) in the β polymorph.

A more detailed description of the crystal structure of the β polymorph, and comparison to the structural properties of the α polymorph, is given in Section S2.†

### Crystal structure prediction of l-tyrosine

To further explore the polymorphic landscape of l-tyrosine, structure prediction calculations were carried out using the AIRSS methodology^45,46^ (see *Methods*). The “initial” geometry optimization in AIRSS produced seven trial crystal structures for further consideration. One pair of trial structures (ranked 1 and 3 in the initial energy ranking using PBE-TS; see Table S1†) resembled the α polymorph and another pair of trial structures (ranked 2 and 6 in the initial energy ranking using PBE-TS; Table S1†) resembled the β polymorph.

The seven trial structures from AIRSS were then subjected to “precise” geometry optimization (using PBE-TS and including relaxation of the unit cell) in FHI-aims, and the results are summarized in Table S2.† In the “precise” geometry optimization, trial structures 1 and 3 converged (with an energy difference of only 0.04 kJ mol^−1^) on the same structure corresponding to the α polymorph, while trial structures 2 and 6 converged (with an energy difference of only 0.06 kJ mol^−1^) on the same structure corresponding to the β polymorph. The good agreement between the crystal structure corresponding to the β polymorph from this calculation (specifically structure 6, which had slightly lower energy than structure 2; see Table S2†) and the crystal structure of the β polymorph determined from Rietveld refinement of powder XRD data is shown in Fig. S7.† Trial structures 4, 5 and 7 represent three distinct polymorphs that have not been reported previously in experimental studies and are hereafter denoted “predicted structures” A, B and C, respectively.

Thus, the seven initial trial structures from AIRSS were reduced to five distinct structures, representing five predicted polymorphs of l-tyrosine. Following the “precise” geometry optimization, the energy of each of the five polymorphs was then determined using a single-point PBE0-MBD calculation. The structure corresponding to the α polymorph is lowest in energy and the structure corresponding to the β polymorph is next lowest in energy. The calculated PBE0-MBD energies of each polymorph, relative to the energy of the α polymorph, are: 4.10 kJ mol^−1^ (β polymorph); 11.11 kJ mol^−1^ (predicted structure A); 11.79 kJ mol^−1^ (predicted structure B); 27.56 kJ mol^−1^ (predicted structure C). As predicted structures A, B and C are all significantly higher in energy than the α polymorph, it is perhaps unlikely^74^ that they represent experimentally accessible meta-stable polymorphs of l-tyrosine. However, the computational approach used here neglects entropic factors, which could have a significant influence on the relative energetic properties of the different structures. Crystallographic data for the five structures predicted by AIRSS (following “precise” geometry optimization) are summarized in Table S3,† and cif files of these structures are included in ESI.†

It is interesting to note that predicted structure A (Fig. S11†) and predicted structure C (Fig. S13†) share several features in common with the β polymorph of l-tyrosine. In particular, these structures have a similar hydrogen-bonded ribbon motif (see Fig. 7) comprising two strands constructed from amino acid head-groups, with N–H⋯O hydrogen bonding between adjacent molecules in a given strand and between molecules in the two strands, and the hydrogen-bonded ribbon is also engaged in hydrogen bonding with the OH groups of molecules involved in adjacent hydrogen-bonded ribbons. The contiguous hydrogen-bonded network in these structures comprises corrugated slabs of l-tyrosine molecules [with mean plane parallel to the *bc*-plane for the β polymorph (Fig. 6) and parallel to the *ab*-plane for predicted structures A (Fig. S11†) and C (Fig. S13†)], which interact with adjacent corrugated slabs through van der Waals interactions. The structures of the β polymorph and predicted structures A and C differ in the details of the topology and the relative arrangement of these corrugated slabs.

In contrast, predicted structure B (Fig. S12†) contains hydrogen-bonded chains of l-tyrosine molecules linked by N–H⋯O interactions between amino acid head-groups (analogous to a single strand of the hydrogen-bonded ribbon motif observed in the β polymorph). Each hydrogen-bonded chain is also engaged in hydrogen bonding with the OH groups of molecules in an adjacent chain. In this structure, the contiguous hydrogen-bonded network comprises columns of molecules (extending along the *a*-axis; Fig. S12†), which interact with adjacent columns through van der Waals interactions.

## Concluding remarks

In conclusion, a new polymorph of l-tyrosine (β polymorph) has been prepared by crystallization from the gas phase, with the crystal structure determined successfully by combined analysis of 3D-ED data and powder XRD data, in conjunction with periodic DFT-D calculations. The final structure of the β polymorph from Rietveld refinement was further validated by: (i) periodic DFT-D geometry optimization, which confirms that the crystal structure is very close to a minimum on the energy landscape, and (ii) comparison between isotropic ^13^C NMR chemical shifts calculated for the crystal structure using DFT-D methodology and those observed in the experimental high-resolution solid-state ^13^C NMR spectrum, which shows an excellent match between calculated and experimental values. Furthermore, DFT-D calculations at the PBE0-MBD level indicate that the crystal structure determined here for the β polymorph is meta-stable relative to the crystal structure of the α polymorph reported previously.

Structure prediction calculations using the AIRSS methodology generated five plausible polymorphic crystal structures for l-tyrosine. The lowest energy structure is the α polymorph, with the β polymorph higher in energy by *ca*. 4 kJ mol^−1^; the other three predicted structures are higher in energy than the α polymorph by more than 11 kJ mol^−1^.

In addition to expanding our understanding of the structural properties of l-tyrosine in the crystalline state, the research strategy described in this paper serves to highlight the significant advantages that can be gained by combined analysis of 3D-ED data and powder XRD data, together with the application of periodic DFT-D calculations and consideration of solid-state NMR data, within a robust protocol for crystal structure determination of materials that are unsuitable for structural characterization by single-crystal XRD.

## Methods

### Sample preparation

The β polymorph of l-tyrosine was prepared by crystallization from the gas phase, following sublimation of a powder sample (*ca*. 12.5 mg) of the α polymorph. The sample was heated under vacuum inside a cylindrical curved-bottom vessel containing a cold finger (see Fig. S1a†). The cold finger was filled with acetone/dry ice at −78 °C (dry ice was added periodically to maintain the temperature at −78 °C). The temperature of the sample was increased from ambient temperature to a target temperature over a period of *ca*. 10 min, and was then left at the target temperature until sublimation was complete. During this time, microcrystalline powder samples deposited on the cold finger and on the outer glass tube (Fig. S1b†). The powder samples collected from these different parts of the apparatus were analyzed separately. In the initial experiments involving sublimation at 300 °C, the sample collected from the outer glass tube was found (from powder XRD analysis) to be a biphasic sample comprising primarily the β polymorph, but with a small amount of the α polymorph. In later experiments involving sublimation at 275 °C, the sample collected from the outer glass tube was found to be a monophasic sample of the β polymorph. In all experiments, the sample collected from the cold finger was a monophasic sample of the α polymorph.

### Powder XRD

Powder XRD data were recorded at ambient temperature (21 °C) on a Bruker D8 Diffractometer (Ge-monochromated CuKα_1_ radiation; Våntec detector covering 3° in 2*θ*; 2*θ* range, 4° to 70°; step size, 0.016°). The polycrystalline sample was packed into a glass capillary (0.7 mm diameter) which was flame sealed and placed on a foil-type sample holder. The data collection time was 64.5 h for the original biphasic sample and 191 h for the pure sample of the β polymorph. Although the powder XRD data were measured in the 2*θ* range from 4° to 70°, no peaks are observed below 2*θ* = 8° and no peaks are predicted for the β polymorph below 2*θ* = 8°. Thus, the region below 2*θ* = 8° was excluded from the powder XRD data used in Pawley fitting and Rietveld refinement.

To assess the degree of preferred orientation in the powder sample, two-dimensional powder XRD data were recorded at ambient temperature on an Agilent SuperNova diffractometer using CuKα radiation and an Atlas detector. The instrument was configured with the two-dimensional detector perpendicular to the incident beam direction, with a sample-to-detector distance of 100 mm. The capillary containing the powder sample was oriented with the capillary axis perpendicular to the incident X-ray beam. Data were collected over a period of 10 s, with the capillary rotated through an angular range of 10° about the capillary axis during the data collection.

### Electron diffraction

The dry powder of the β polymorph of l-tyrosine was dispersed directly on a continuous carbon film supported by a 200-mesh copper grid, which was then mounted on a Gatan cryo-transfer tomography holder (No. 914). 3D-ED data were collected using a JEOL JEM-2100 transmission electron microscope (TEM) operating at an accelerating voltage of 200 kV, with the sample cooled to *ca*. 100 K. The 3D-ED data were recorded using the continuous rotation electron diffraction (cRED) method^51^ by continuously tilting the goniometer with tilt speed 0.454° per second. During tilting, the crystal was tracked by sequential defocusing of the intermediate lens using the Instamatic software.^75^ The data collection covered a rotation range of 98.41° (for more details, see Table S4†). The diffraction patterns were collected using the high-speed hybrid detection camera Timepix Quad (ASI) with integration time 0.5 s. The datasets were processed using X-ray Detector Software (XDS)^52^ in order to extract intensity data. Structure refinement against 3D-ED data was carried out using SHELXL,^76^ with isotropic atomic displacement parameters and atomic scattering factors for electrons. Hydrogen atoms were included within the structural model based on geometric restraints using the HFIX command in SHELXL.

### Solid-state NMR spectroscopy

High-resolution solid-state ^13^C NMR data were recorded at ambient temperature (20 °C) for samples of the α polymorph and the β polymorph of l-tyrosine on a Bruker AVANCE III spectrometer at the U. K. High-Field (850 MHz) Solid-State NMR Facility (^13^C Larmor frequency, 213.8 MHz; 4 mm HX probe; zirconia rotor; MAS frequency, 12 kHz) using ramped ^1^H → ^13^C cross-polarization with ^1^H decoupling (using SPINAL-64) applied during acquisition. The total number of scans acquired was 32 for the α polymorph and 224 for the β polymorph, with a recycle delay of 60 s between each scan. The ^13^C NMR data were referenced using l-alanine, for which the carboxylate resonance was set to 177.9 ppm.

### DFT-D calculations in conjunction with structure determination of the β polymorph from powder XRD data

Periodic DFT-D calculations were carried out at various stages of the structure determination of the β polymorph (particularly in conjunction with Rietveld refinement from powder XRD data) using the CASTEP program^77^ (Academic Release Version 8.0). In particular, structural models were subjected to geometry optimization (with fixed unit cell) using ultrasoft pseudopotentials,^78^ PBE functional,^79^ semi-empirical dispersion correction using the TS correction scheme,^80^ preserved space group symmetry, periodic boundary conditions, a basis set cut-off energy of 700 eV and a Monkhorst–Pack grid^81^ of minimum sample spacing (0.05 × 2π) Å^−1^. Convergence criteria for geometry optimization were 0.01 eV Å^−1^ for the maximum atomic force, 0.00001 eV per atom on the total energy, and 0.001 Å for atomic displacements.

### DFT-D calculations of solid-state ^13^C NMR data

Solid-state ^13^C NMR chemical shifts were calculated using CASTEP (Academic Release version 8.0) for the crystal structure of the β polymorph of l-tyrosine obtained in the Rietveld refinement and following DFT-D geometry optimization (with fixed unit cell) of this structure, as described above. The same method was used to calculate the solid-state ^13^C NMR chemical shifts for the published crystal structure^47^ of the α polymorph of l-tyrosine. In these calculations, the Gauge Including Projector Augmented Wave (GIPAW) approach^82–87^ was used with a cut-off energy of 700 eV and PBE functional. A set of isotropic ^13^C NMR shielding values was generated from the CASTEP calculations. From the isotropic ^13^C NMR shielding value (*σ*_calc_) calculated for each ^13^C environment in the crystal structure, the corresponding calculated isotropic ^13^C NMR chemical shift (*δ*_calc_) was determined^84^ from the equation *δ*_calc_ = 〈*δ*_exp_〉 + 〈*σ*_calc_〉 − *σ*_calc_, where 〈*δ*_exp_〉 denotes the mean of the isotropic ^13^C NMR chemical shifts determined from the experimental high-resolution solid-state ^13^C NMR spectrum and 〈*σ*_calc_〉 denotes the mean of the calculated isotropic ^13^C NMR shielding values. For the β polymorph, 〈*δ*_exp_〉 = 116.30 ppm and 〈*σ*_calc_〉 = 54.21 ppm. For the α polymorph, 〈*δ*_exp_〉 = 116.23 ppm and 〈*σ*_calc_〉 = 53.33 ppm.

### DFT-D calculations to compare the energetic properties of the α and β polymorphs of l-tyrosine

After completing the Rietveld refinement, comparison of the energetic properties of the β polymorph and the previously reported α polymorph was carried out through DFT-D calculations using the FHI-aims software package^88^ (date stamp: 191029). In this component of the work, different functionals and different methods for dispersion correction were considered, with FHI-aims selected for this study on account of its efficiency for periodic hybrid-DFT calculations.^89,90^ Initially, the final refined crystal structure of the β polymorph from the Rietveld refinement and the previously published crystal structure of the α polymorph^47^ were subjected to geometry optimization with fixed unit cell using the PBE-TS method, and based on a convergence criterion of 0.01 eV Å^−1^ for the maximum atomic force. Following the geometry optimization, single-point energy calculations were carried out using both generalized gradient approximation (GGA) and hybrid-GGA exchange-correlation functionals, specifically PBE^79^ and PBE0,^91^ coupled with either the Tkatchenko–Scheffler (TS) method^80^ or the many-body dispersion (MBD) method^92^ for dispersion correction. Thus, the complete set of exchange-correlation functionals considered in the single-point energy calculations were: PBE-TS, PBE-MBD, PBE0-TS and PBE0-MBD. All calculations were carried out with an “intermediate” basis set and relativistic effects were included *via* the scaled zeroth order regular approximation.^88^ A Γ-centred **k**-grid was used with a minimum sample spacing of (0.05 × 2π) Å^−1^ [testing with a denser **k**-grid sampling of (0.04 × 2π) Å^−1^ gave changes in relative energies of less than 1 meV]. The electronic structure self-consistent field (SCF) cycle was considered to be converged when changes in the electron density, the total energy and the sum of the eigenvalue energies were below 10^−6^ e a_0_^−3^, 10^−6^ eV and 10^−6^ eV, respectively.

### Crystal structure prediction

Crystal structure prediction of l-tyrosine was carried out using the *ab initio* random structure searching (AIRSS) method,^45,46^ which has recently been used successfully for other organic molecular crystals.^93,94^ The AIRSS methodology for structure prediction involves two steps: (1) generation of trial structures by “intelligent” random searching, and (2) geometry optimization and energy-ranking of the trial structures using full periodic DFT calculations (PBE-TS).

Stage (1) considered crystal structures containing *Z* molecules per unit cell, with *Z* = 1, 2, 3, 4 or 6. Structures were generated from an initial, randomly placed molecule by applying the symmetry operators of all space groups. In this structure-generation step, the unit cell parameters were allowed to vary within constraints to give a volume per molecule within 10% of an initial estimate of 210 Å^3^. Minimum intermolecular atom–atom distances were also specified in order to eliminate structures containing unreasonable intermolecular contacts. Searches with smaller unit cells (corresponding to *Z* = 1 or 2) were carried out with a global minimum separation of 2 Å. The minimum separations between intermolecular pairs of atomic species in the resulting low-energy structures were determined and used to specify minimum separations in searches with larger unit cells (corresponding to *Z* = 3, 4 or 6). In stage (2), “initial” geometry optimization (including relaxation of the unit cell) and energy ranking was achieved by DFT calculations using the CASTEP code,^77^ which uses a plane-wave basis-set together with pseudopotentials to represent the core–valence interaction. All calculations used the PBEsol functional,^95^ a plane-wave cut-off energy of 800 eV and a Brillouin zone sampling of (0.07 × 2π) Å^−1^. The convergence criteria were set to the CASTEP default tolerances of 2 × 10^−5^ eV per atom on the total energy, 0.05 eV Å^−1^ on the maximum atomic force, 1 × 10^−3^ Å on the maximum atomic displacement, and 0.1 GPa on the maximum stress component. This initial structure prediction produced low-energy structures only for *Z* = 2 and *Z* = 4. Structures in non-chiral space groups were removed from the initial structure prediction list, giving only the chiral structures of interest. The relative energies of the predicted crystal structures generated by the two steps of the AIRSS methodology are shown in Table S1.†

Each of the predicted crystal structures resulting from the two stages of AIRSS was then examined by further DFT-D calculations using FHI-aims. First, a “precise” geometry optimization (including relaxation of unit cell parameters) was carried out on each structure using the PBE-TS method and based on a convergence criterion of 0.01 eV Å^−1^ for the maximum atomic force. Second, for each structure obtained following the “precise” geometry optimization, a single-point calculation was carried out using the PBE0-MBD method to obtain a more reliable energy ranking.^72,73^ The results of the energy ranking of the predicted crystal structures following the “precise” geometry optimization and the subsequent single-point PBE0-MBD calculations are given in Table S2.†

### Comparison of crystal structures

All overlay plots for comparison of crystal structures (Fig. 2 and S5–S7†) were generated in Mathematica (Version 12.3).^96^ In each case, the origins of the unit cells of the two structures were fixed at the same point, the *a*-axes of the two structures were aligned parallel to each other, and the *b*-axes of the two structures were aligned parallel to each other (we note that, for the monoclinic system, the *a*-axis and *b*-axis are orthogonal). For the overlay plots comparing structures with slightly different unit cells (Fig. 2 and S7†), both unit cells are shown (as indicated in the figure captions).

## Data availability

Supporting Information contains: (i) additional figures discussed in the text, (ii) a detailed description of the crystal structure of the β polymorph of l-tyrosine and comparison to the α polymorph, (iii) tables of results from AIRSS calculations for crystal structure prediction of l-tyrosine, (iv) definition of *R*-factors used in direct-space structure solution from 3D-ED data and powder XRD data, (v) 3D-ED data statistics, (vi) computed values of isotropic ^13^C NMR chemical shifts for the α and β polymorphs of l-tyrosine, (vii) crystallographic information (cif) file for the crystal structure of the β polymorph of l-tyrosine determined in the final Rietveld refinement from powder XRD data, and (viii) crystallographic information files for the five distinct crystal structures of l-tyrosine generated from the AIRSS structure prediction calculations (specifically, the predicted structure corresponding to the α polymorph, the predicted structure corresponding to the β polymorph, and the new predicted structures A, B and C).

Data and results from experimental and computational studies (with the exception of the DFT-D calculations using FHI-Aims) are available at: https://doi.org/10.17035/d.2022.0164412918. Results from DFT-D calculations using FHI-Aims are available at: https://doi.org/10.17172/NOMAD/2021.10.18-1.

The crystal structure of the β polymorph of l-tyrosine determined in the final Rietveld refinement from powder XRD data is also available at CCDC 2114085.

## Author contributions

The research project was coordinated by K. D. M. H., with specific components of the research carried out as follows: preparation of the sample of the new polymorph of l-tyrosine (H. E. H., C. E. H.); measurement of powder XRD data (H. E. H., C. J. H. S.); measurement, analysis and interpretation of 3D-ED data, including unit cell determination (D. N. J., T. W., P. A. M.); structure solution (using EAGER) and structure refinement from powder XRD data (C. J. H. S., M. T. Y., C. E. H.); structure solution (using EAGER) from 3D-ED data (C. J. H. S.); structure refinement from 3D-ED data (T. W.); measurement of solid-state NMR data (C. E. H.); analysis of solid-state NMR data (C. E. H., C. J. H. S.); structure prediction by AIRSS calculations (C. J. P.); DFT-D calculations using FHI-Aims to assess the relative energies of polymorphs (A. J. L.). Preparation of the manuscript was coordinated by K. D. M. H. with contributions from all authors.

## Conflicts of interest

There are no conflicts to declare.

## Supplementary Material

SC-013-D1SC06467C-s001

SC-013-D1SC06467C-s002
