# Supplementary material for: A structure determination protocol based on combined analysis of 3D-ED data, powder XRD data, solid-state NMR data and DFT-D calculations reveals the structure of a new polymorph of l-tyrosine
Source: Chem Sci. 2022 Mar 30;13(18):5277–88. doi: 10.1039/d1sc06467c (PMC9093151; doi:10.1039/d1sc06467c)

Electronic Supplementary Material (ESI) for Chemical Science.
This journal is © The Royal Society of Chemistry 2022

Electronic Supplementary Information

A structure determination protocol based on combined analysis of 3D-ED data, powder XRD data, solid-state NMR data and DFT-D calculations reveals the structure of a new polymorph of L-tyrosine

Christopher J. H. Smalley,[1] Harriet E. Hoskyns,[1] Colan E. Hughes,[1] Duncan N. Johnstone,[2] Tom Willhammar,[3] Mark T. Young,[4] Christopher J. Pickard,[2,5] Andrew J. Logsdail,[6] Paul A. Midgley,[2] Kenneth D. M. Harris[1]*

1 School of Chemistry, Cardiff University, Park Place, Cardiff CF10 3AT, Wales, U.K.
2 Department of Materials Science, University of Cambridge, 27 Charles Babbage Road, Cambridge CB3 0FS, England, U.K.
3 Department of Materials and Environmental Chemistry, Stockholm University, Svante Arrhenius väg 16C, 106 91 Stockholm, Sweden
4 School of Biosciences, Cardiff University, Cardiff CF10 3AX, Wales, U.K.
5 Advanced Institute for Materials Research, Tohoku University 2-1-1 Katahira, Aoba, Sendai, 980-8577, Japan
6 Cardiff Catalysis Institute, School of Chemistry, Cardiff University, Park Place, Cardiff CF10 3AT, Wales, U.K.

* Author for correspondence: HarrisKDM@cardiff.ac.uk

Section S1 Additional Figures

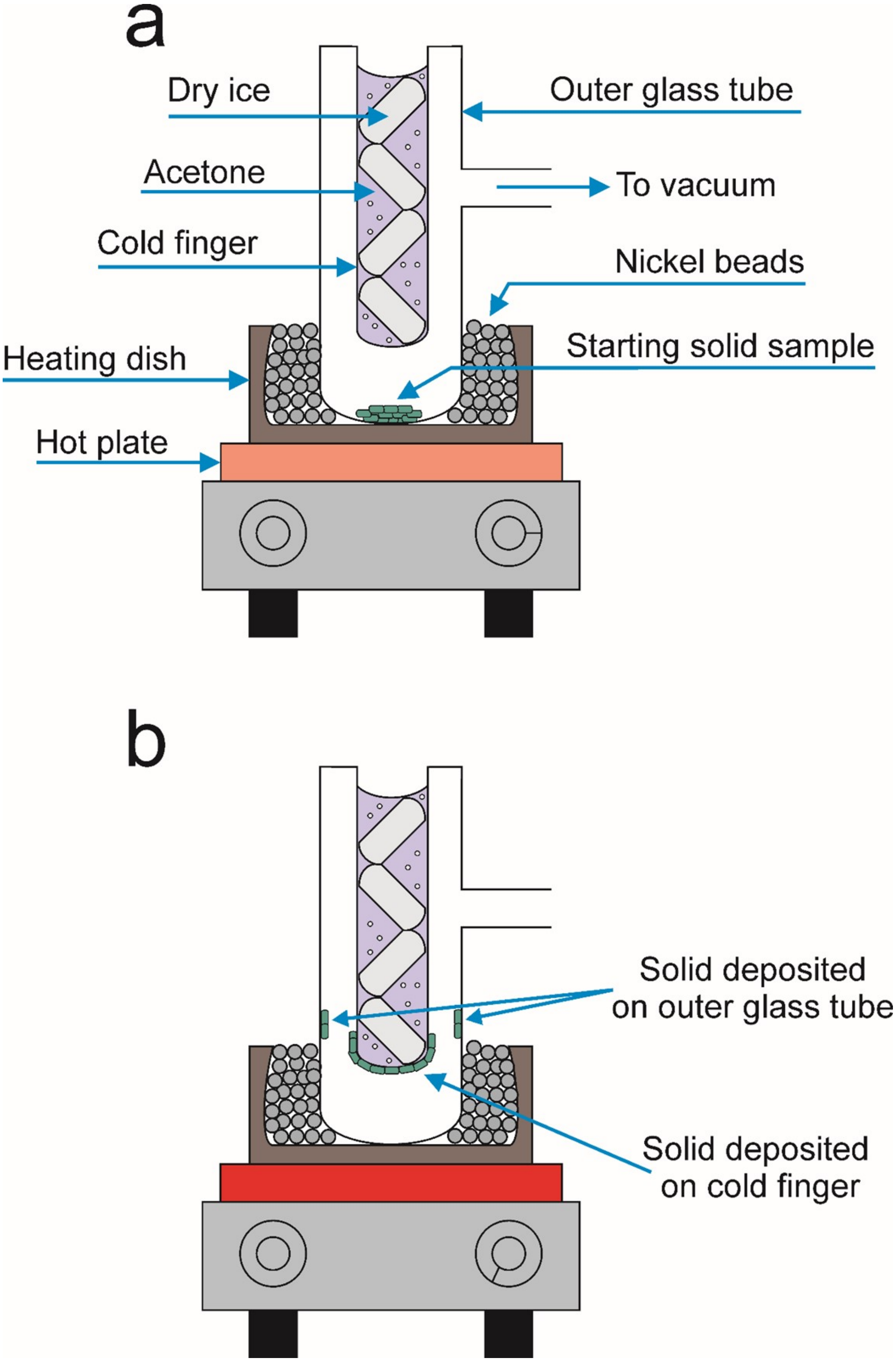

Figure S1. Schematic of the experimental apparatus for crystallization from the gas phase: (a) the experimental set up before sublimation of the original solid sample, and (b) the experimental set-up after sublimation of the original solid sample, with crystallization occurring both on the cold finger and on the outer glass tube.

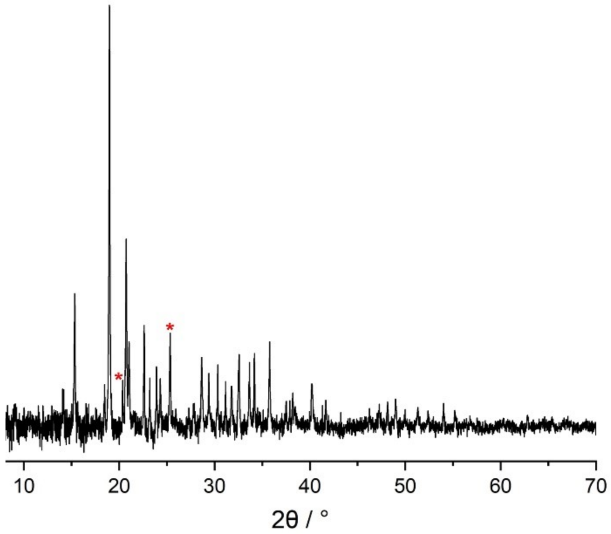

Figure S2. Powder XRD pattern of the initial biphasic sample containing the new β polymorph of L-tyrosine. Following successful unit cell determination of the β polymorph from 3D-ED data, it was clear from the powder XRD data that the biphasic sample comprised predominantly the β polymorph together with a small amount of a second phase, identified as the α polymorph of L-tyrosine (the main peaks due to the α polymorph are indicated by red asterisks).

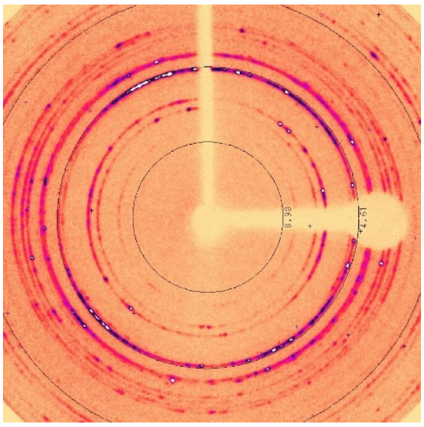

Figure S3. Powder XRD data recorded for the monophasic sample of the β polymorph of L-tyrosine using a two-dimensional detector, showing a non-uniform distribution of intensities on the Debye-Scherrer rings.

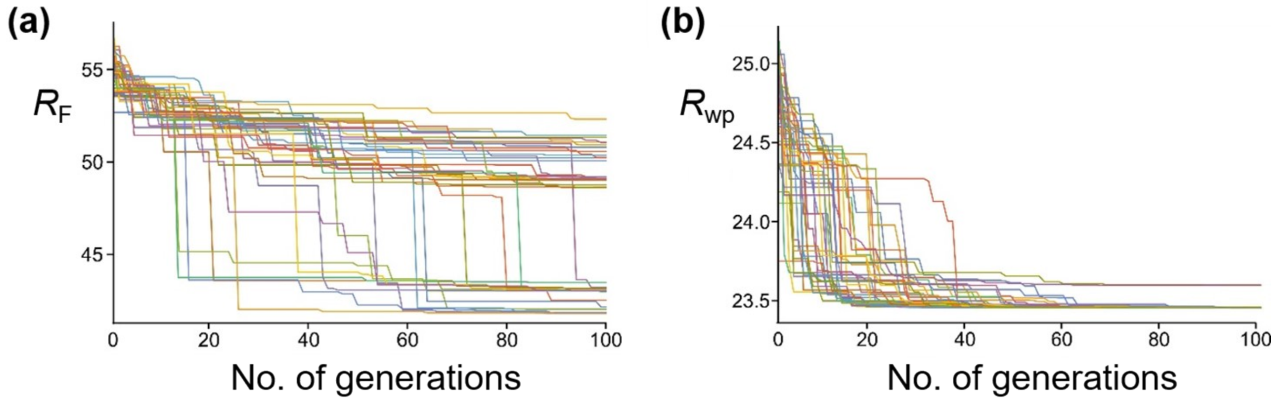

Figure S4. Evolutionary progress in the GA structure solution calculations using (a) the 3D-ED data and (b) the powder XRD data for the original biphasic sample. In each case, the evolution is shown for 40 independent GA calculations (each starting from a different random initial population of trial structures). Each continuous line represents the evolution of one of the 40 independent GA calculations and shows the lowest value of *R*-factor among all 100 trial structures in the population as a function of generation number. The *R*-factors used in the analysis of the 3D-ED data (R_F) and the powder XRD data (R_{wp}) are defined in Section S3 (note that that the absolute values of R_F and R_{wp} cannot be compared directly). After 100 generations, the success rate in finding the correct structure solution is significantly higher for the powder XRD data than the 3D-ED data. For the 3D-ED data, 7 of the 40 independent GA calculations generated essentially the same structure solution with low *R*-factor; these 7 structures are among those with R_F in the range 41.8% – 43.2% shown in (a). For the powder XRD data, 38 of the 40 independent GA calculations generated essentially the same structure solution with lowest *R*-factor, corresponding to those with $R_{wp} \approx 23.5\%$ shown in (b).

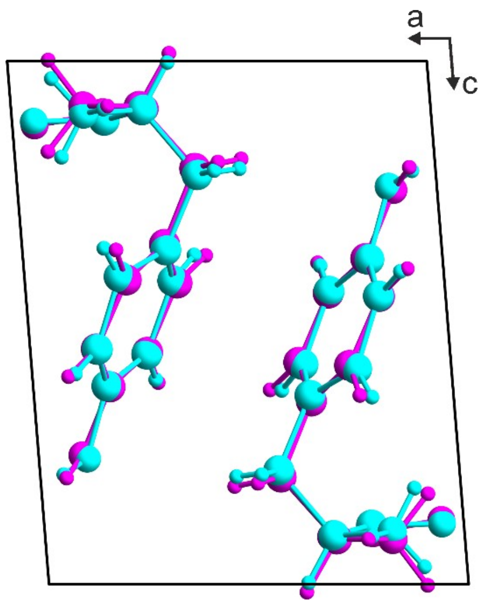

Figure S5. Overlay of the crystal structures (viewed along the *b*-axis) of the β polymorph of L-tyrosine obtained by Rietveld refinement from the powder XRD data (magenta) and by refinement from the 3D-ED data (cyan).

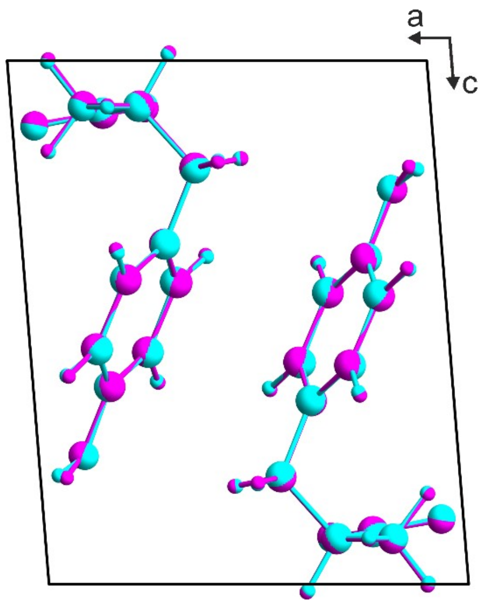

Figure S6. Overlay of the crystal structure of the β polymorph of L-tyrosine obtained in the final Rietveld refinement (magenta) and the crystal structure obtained after subjecting this structure to periodic DFT-D geometry optimization using PBE-TS with fixed unit cell (cyan).

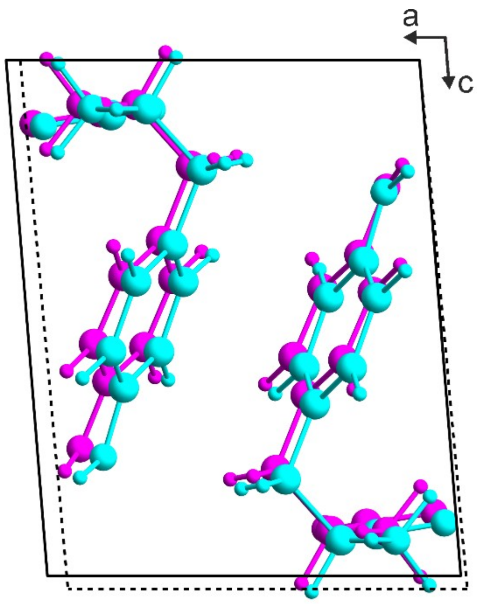

Figure S7. Overlay of the crystal structure of the β polymorph of L-tyrosine from the final Rietveld refinement (magenta; unit cell shown by the solid black lines) and the predicted crystal structure corresponding to the β polymorph generated by AIRSS followed by "precise" geometry optimization including unit cell relaxation (cyan; unit cell shown by the dashed black lines).

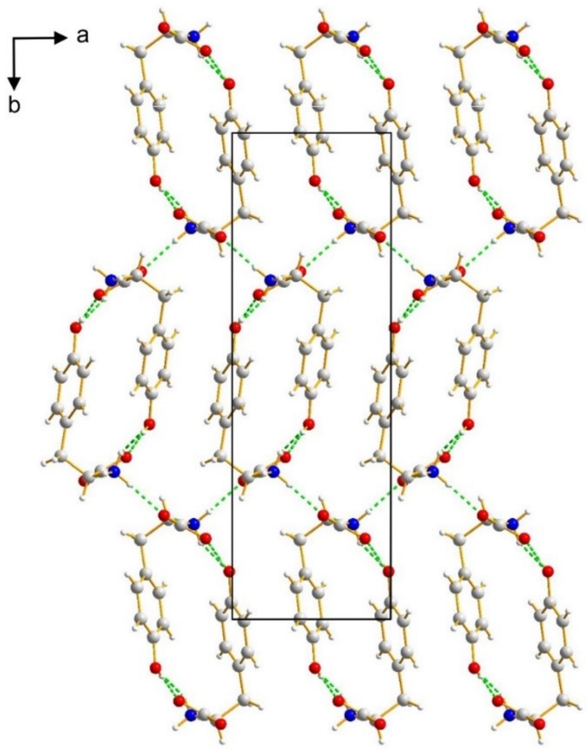

Figure S8. Crystal structure of the α polymorph of L-tyrosine (determined previously: A. Mostad, H. M. Nissen, C. Romming, *Acta Chem. Scand*. **1972**, *26*, 3819-3833) viewed along the *c*-axis. The structure comprises alternating hydrophilic and hydrophobic layers parallel to the *ac*-plane. Hydrogen bonds are indicated by green dashed lines.

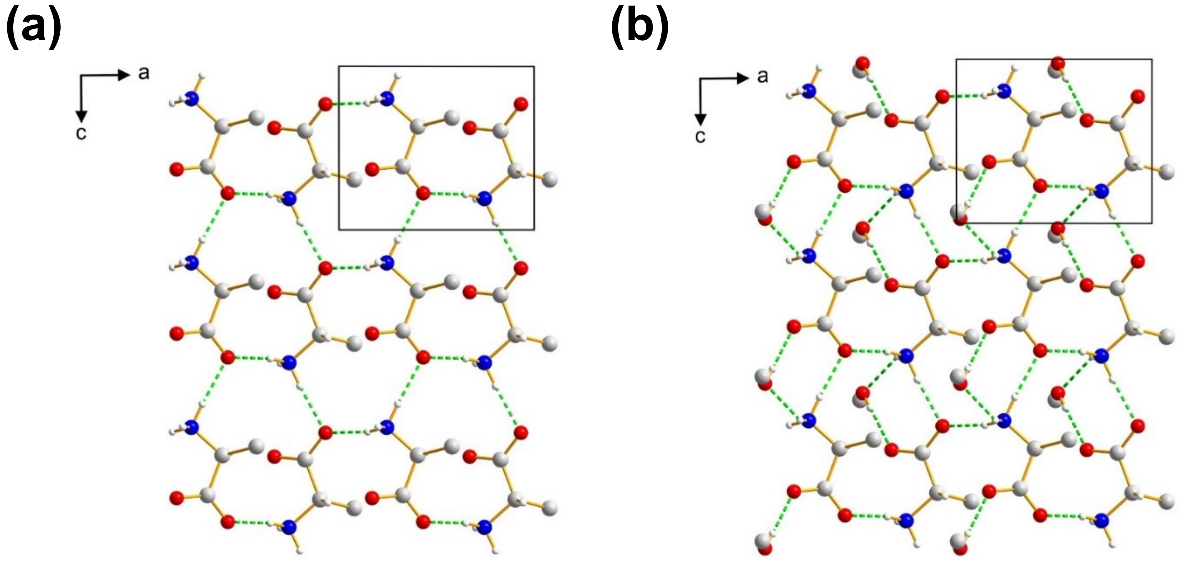

Figure S9. The two-dimensional hydrogen-bonding arrangement in the hydrophilic layer of the crystal structure of the α polymorph of L-tyrosine (determined previously: A. Mostad, H. M. Nissen, C. Romming, *Acta Chem. Scand*. **1972**, *26*, 3819-3833) viewed along the *b*-axis, showing: (a) only the amino acid head-groups, and (b) both the amino acid head-groups and the OH groups of the side-chains. For clarity, only the $CCH(NH_3^+)CO_2^-$ unit of each head-group and the COH unit of each side-chain are shown. Hydrogen bonds are indicated by green dashed lines.

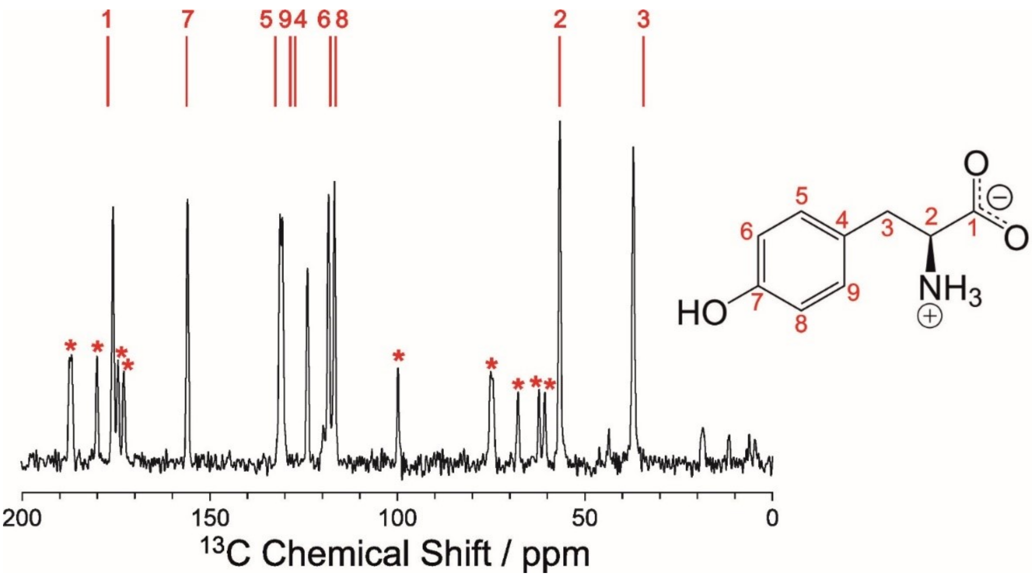

Figure S10. Experimental high-resolution solid-state ^{13}C NMR spectrum recorded for the α polymorph of L-tyrosine together with the values of isotropic ^{13}C NMR chemical shifts calculated for the published crystal structure of the α polymorph (indicated by the red lines above the spectrum). The specific ^{13}C site corresponding to each calculated value is indicated. Spinning sidebands in the experimental spectrum are marked by red asterisks.

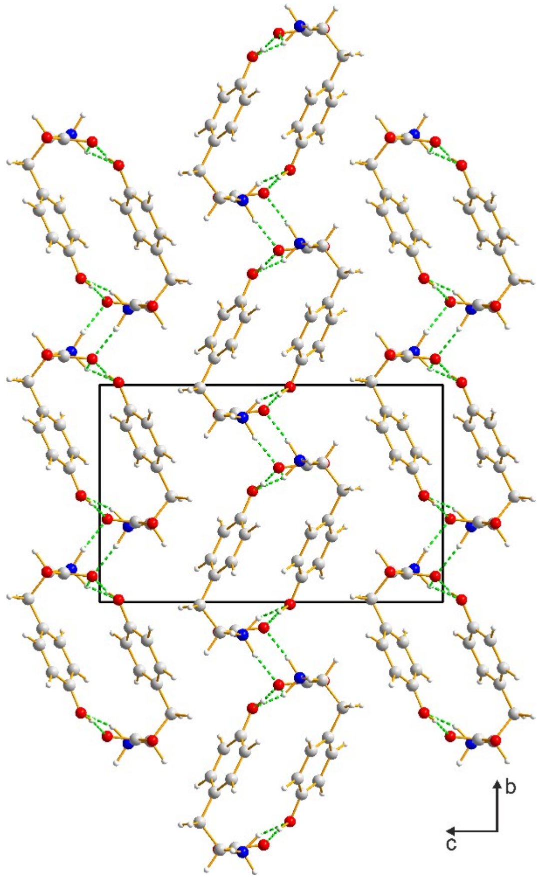

Figure S11. Predicted structure A of L-tyrosine (generated by AIRSS, followed by "precise" geometry optimization) viewed along the *a*-axis. Hydrogen bonds are indicated by green dashed lines.

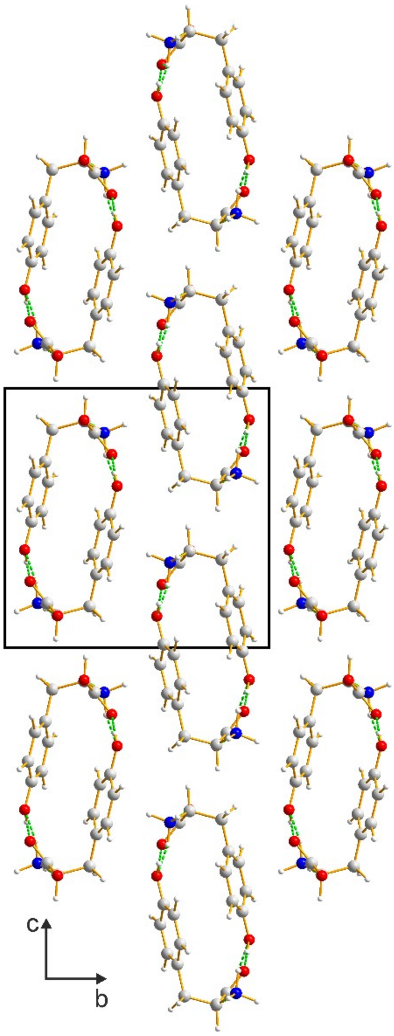

Figure S12. Predicted structure B of L-tyrosine (generated by AIRSS, followed by "precise" geometry optimization) viewed along the *a*-axis. Hydrogen bonds are indicated by green dashed lines.

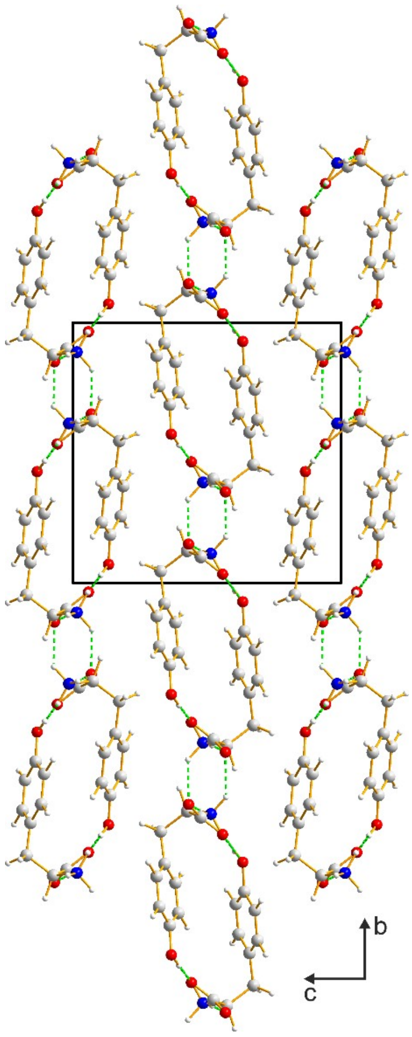

Figure S13. Predicted structure C of L-tyrosine (generated by AIRSS, followed by "precise" geometry optimization) viewed along the *a*-axis. Hydrogen bonds are indicated by green dashed lines.

Section S2 Detailed Description on the Crystal Structure of the β Polymorph of L-Tyrosine, and Comparison to the α Polymorph

The β polymorph of L-tyrosine (Figure 6) may be described as a bilayer structure, comprising alternate hydrophobic and hydrophilic layers parallel to the *ab*-plane. The hydrophilic region contains the amino acid head-groups and the OH groups of the side-chains, while the hydrophobic region contains the phenyl rings of the side-chains. The hydrogen-bonding involving the amino acid head-groups comprises a ribbon motif (Figure 7), propagating along the *b*-axis, and constructed from two strands of L-tyrosine molecules. Within a given strand, adjacent molecules are related by translation along the *b*-axis, while the two strands are related to each other by the 2_1 screw along the *b*-axis. In each molecule, the atoms of the N–C(α)–CO_2 unit are essentially co-planar, and this plane is essentially parallel to the crystallographic *ab*-plane. Within a given hydrogen-bonded ribbon, the planes of the N–C(α)–CO_2 units of the molecules in each strand are parallel to each other, but displaced slightly along the *c*-axis (Figure 8). The hydrogen-bonded ribbon (Figure 7) is constructed from short and relatively linear N–H···O hydrogen bonds, both between adjacent molecules in a given strand (N···O, 2.70 Å; N–H···O, 178.4°) and between molecules in the two strands (N···O, 2.79 Å; N–H···O, 159.1°), giving rise to a cyclic hydrogen-bonded array described as $R_3^3(11)$ in graph set notation. Significantly, a given hydrogen-bonded ribbon is not engaged in hydrogen bonding with any other ribbon. However, the hydrogen-bonded ribbon is involved in additional hydrogen bonding with the OH groups of the side-chains of the molecules that form the hydrogen-bonded ribbons in the layers "above" and "below" along the *c*-axis. As shown in Figure 8, each OH group serves as the donor in an O–H···O hydrogen bond (O···O, 2.62 Å; O–H···O, 165.2°) and as the acceptor in an N–H···O hydrogen bond (N···O, 2.86 Å; N–H···O, 129.7°) with carboxylate and ammonium groups, respectively, in the hydrogen-bonded ribbon, giving a cyclic motif described as $R_3^3(8)$ in graph set notation.

We now compare the structural properties of the α and β polymorphs of L-tyrosine, firstly noting that the molecular conformations (defined by torsion angles τ_1, τ_2 and τ_3; see Figure 1) are similar in each case: τ_1 (O–C–C–C) = –71.06° (α), –62.78° (β); τ_2 (C–C–C–C)= –53.08° (α), –53.99° (β); τ_3 (C–C–C–C) = 95.51° (α), 99.96° (β). However, in contrast to the one-dimensional hydrogen-bonded ribbons propagating along the *b*-axis in the β polymorph, the hydrophilic region of the α polymorph is a two-dimensional hydrogen-bonded array (parallel to the *ac*-plane; see Figures S8 and S9) involving the amino acid head-groups and the OH groups of the side-chains of L-tyrosine

molecules. As a consequence, the α polymorph is a three-dimensionally connected hydrogen-bonded structure, whereas the contiguous hydrogen-bonded network in the β polymorph comprises corrugated slabs with a mean plane parallel to the *bc*-plane (Figure 6), constructed from the hydrogen-bonded ribbons parallel to the *b*-axis and hydrogen-bonded linkages (involving the OH groups) to the adjacent ribbons along the *c*-axis. Adjacent corrugated slabs in the β polymorph are related by translation along the *a*-axis and "nestle" into each other through van der Waals interactions, with no hydrogen-bonding interactions between adjacent corrugated slabs.

Section S3 Tables of Results from AIRSS Calculations for Crystal Structure Prediction of L-Tyrosine

Table S1. Relative energy (Δ*E*, expressed per mole of L-tyrosine molecules) for the seven predicted crystal structures of L-tyrosine generated by AIRSS. For each crystal structure generated by AIRSS, the single-point PBE-TS energy was calculated using FHI-aims. The values of Δ*E* are given relative to crystal structure 1 (the structure of lowest energy generated by AIRSS).

Structure from AIRSS	Δ*E* (PBE-TS) / kJ mol^{-1} (single-point calculation)
1	0.00
2	6.03
3	6.64
4	14.58
5	15.79
6	18.65
7	17.92

Table S2. Relative energy (Δ*E*, expressed per mole of L-tyrosine molecules) after subjecting the crystal structures of L-tyrosine generated by AIRSS to "precise" geometry optimization (including relaxation of unit cell parameters and nuclear coordinates) using PBE-TS in FHI-aims. After geometry optimization, structures 1 and 3 converge on an equivalent structure corresponding to the experimentally observed α polymorph, and structures 2 and 6 converge on an equivalent structure corresponding to the experimentally observed β polymorph. In the manuscript, results are presented for the *more stable* of these structures for each polymorph (i.e., structure 3 for the α polymorph and structure 6 for the β polymorph). Values of Δ*E* are expressed relative to the α polymorph (i.e., structure 3). The single-point PBE0-MBD energy was also calculated using FHI-aims for each structure following the "precise" geometry optimization, giving the high-accuracy relative energies reported in the manuscript. The crystal structures of the α polymorph (structure 3), β polymorph (structure 6), predicted structure A (structure 4), predicted structure B (structure 5) and predicted structure C (structure 7) following the "precise" geometry optimization are included as cif files in Electronic Supplementary Information.

Structure from AIRSS	Assignment	Δ*E* (PBE-TS) / kJ mol^{-1} (after geometry optimization)	Δ*E* (PBE0-MBD) / kJ mol^{-1} (single-point calculation)
3	α polymorph	0.00	0.00
1	α polymorph	0.02	0.04
6	β polymorph	4.38	4.10
2	β polymorph	4.41	4.16
4	Predicted structure A	11.69	11.11
5	Predicted structure B	13.28	11.83
7	Predicted structure C	16.07	27.56

Table S3. Crystallographic data for the five distinct structures of L-tyrosine generated from the AIRSS structure prediction calculations, following "precise" geometry optimization (including relaxation of unit cell parameters). The structures labelled as the α polymorph and β polymorph correspond to the experimentally determined crystal structures of these polymorphs. The structures labelled as the predicted structures A, B and C have not been observed in experimental studies.

Structure	Space Group	Z	a / Å	b / Å	c / Å	α / °	β / °	γ / °	V / Å^3
α polymorph	$P2_12_12_1$	4	5.86	21.13	6.80	90	90	90	841.99
β polymorph	$P2_1$	2	7.41	5.90	9.87	90	95.15	90	429.76
Predicted structure A	$P2_12_12_1$	4	5.90	9.75	15.39	90	90	90	885.31
Predicted structure B	$P2_12_12_1$	4	5.92	12.08	11.88	90	90	90	849.58
Predicted structure C	$P2_12_12_1$	4	5.87	11.80	12.13	90	90	90	840.20

Section S4 Definition of *R*-factors used in Direct-Space Structure Solution from 3D-ED data and Powder XRD Data

For powder XRD data, the weighted profile *R*-factor (R_{wp}) and the unweighted profile *R*-factor (R_p) are defined as follows:

$$R_{wp} = 100 \times \left(\frac{\sum_i w_i (y_{o,i} - y_{c,i})^2}{\sum_i w_i (y_{o,i})^2} \right)^{1/2} \quad \text{(S1)}$$

$$R_p = 100 \times \left(\frac{\sum_i (y_{o,i} - y_{c,i})^2}{\sum_i (y_{o,i})^2} \right)^{1/2} \quad \text{(S2)}$$

where $y_{o,i}$ is the intensity of the *i*th data point in the digitized experimental powder XRD pattern, $y_{c,i}$ is the intensity of the *i*th data point in the digitized powder XRD pattern calculated for the structural model, and w_i is a weighting factor for the *i*th data point, given by $w_i = 1/y_{o,i}$.

For 3D-ED data, the *R*-factor (R_F) is defined as:

$$R_F = 100 \times \left(\frac{\sum_i \left| |F_{o,i}| - |F_{c,i}| \right|}{\sum_i |F_{o,i}|} \right) \quad \text{(S3)}$$

where $|F_{o,i}|$ is the structure factor amplitude (the square-root of the measured intensity) for the *i*th reflection in the experimental 3D-ED dataset and $|F_{c,i}|$ is the structure factor amplitude for the corresponding reflection calculated for the structural model.

Section S5 3D-ED Data Statistics

Table S4: 3D electron diffraction (3D-ED) data for the β polymorph of L-tyrosine.

Crystal system	Monoclinic
Space group	$P2_1$ (no. 4)
a / Å	7.92
b / Å	6.13
c / Å	9.90
α / °	90
β / °	94.82
γ / °	90
V / Å^3	478.9
λ / Å	0.0251
Exposure time per frame / s	0.5
Tilt speed / ° s^{-1}	0.2321
Completeness / %	49.0
Resolution / Å	0.85
R_{int}	0.112
No. of symmetry independent reflections	702
No. of refined parameters	53
No. of restraints	12
Refinement R-value	0.251

<u>Section S6</u> High-resolution Solid-state ^{13}C NMR Spectroscopy

Table S5. Isotropic ^{13}C NMR chemical shifts (δ_{calc}) calculated for the crystal structures of the α and β polymorphs of L-tyrosine, with the numbering of the ^{13}C sites defined in the figure below. Figure 5 of the main text shows the experimental high-resolution solid-state ^{13}C NMR spectrum for the β polymorph and Figure S10 shows the experimental high-resolution solid-state ^{13}C NMR spectrum for the α polymorph. In each figure, the calculated values of the isotropic ^{13}C NMR chemical shifts (given in this table) are shown above the experimental spectrum for comparison.

^{13}C site	δ_{calc} / ppm	
	β polymorph	α polymorph
1	177.93	179.47
2	54.45	55.99
3	36.95	38.49
4	124.65	126.19
5	132.05	133.59
6	117.91	119.45
7	157.89	159.43
8	113.41	114.95
9	131.43	132.97

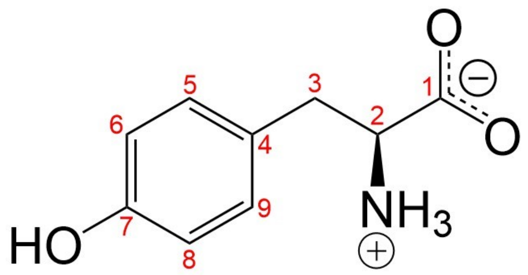

Supplement: SC-013-D1SC06467C-s001 [file SC-013-D1SC06467C-s001.pdf]
